# A new on-axis micro-spectrophotometer for combining Raman, fluorescence and UV/Vis absorption spectroscopy with macromolecular crystallography at the Swiss Light Source

**DOI:** 10.1107/S0909049513016063

**Published:** 2013-07-05

**Authors:** Guillaume Pompidor, Florian S. N. Dworkowski, Vincent Thominet, Clemens Schulze-Briese, Martin R. Fuchs

**Affiliations:** a Paul Scherrer Institut, CH-5232 Villigen, Switzerland; b DECTRIS Ltd, CH-5400 Baden, Switzerland

**Keywords:** macromolecular crystallography, single-crystal spectroscopy, micro-spectrophotometry, complementary techniques, Raman spectroscopy

## Abstract

The combination of X-ray diffraction experiments with optical methods such as Raman, UV/Vis absorption and fluorescence spectroscopy greatly enhances and complements the specificity of the obtained information. The upgraded version of the *in situ* on-axis micro-spectrophotometer, MS2, at the macromolecular crystallography beamline X10SA of the Swiss Light Source is presented. The instrument newly supports Raman and resonance Raman spectroscopy, in addition to the previously available UV/Vis absorption and fluorescence modes. With the recent upgrades of the spectral bandwidth, instrument stability, detection efficiency and control software, the application range of the instrument and its ease of operation were greatly improved. Its on-axis geometry with collinear X-ray and optical axes to ensure optimal control of the overlap of sample volumes probed by each technique is still unique amongst comparable facilities worldwide and the instrument has now been in general user operation for over two years.

## Abstract 2

The new version MS2 of the *in situ* on-axis micro-spectrophotometer at the macromolecular crystallography beamline X10SA of the Swiss Light Source supports the concurrent acquisition of Raman, resonance Raman, fluorescence and UV/Vis absorption spectra along with diffraction data.

## 1. Introduction

By combining macromolecular crystallography (MX) with optical spectroscopic techniques the structural biologist is provided with a wide array of tools to extend what can be learned from the crystallographic structure alone. By resolving critical ambiguities in the interpretation of the experimentally obtained electron density maps, the scope and capacity of MX can be significantly extended in several fields relevant to structural biology, such as kinetic enzymology or ligand binding studies. For example, the assignments provided by optical spectroscopy are a widely used tool for structural enzymology to characterize freeze-trapped reaction intermediates (Schlichting *et al.*, 2000 ▶; Weik & Colletier, 2010 ▶), and thereby give access to kinetic reaction studies in the crystal.

Optical spectroscopy extends the parameters that are experimentally accessible from a protein crystal by providing specific information on sample properties like electronic state, bond strength and coordination. This independent experimental observation of additional parameters can be essential in asserting the chemical identity of key sites in a macromolecule like, for example, a ligand, a co-factor or a functionally important amino acid residue.

These new opportunities have begun to be seized by the crystallographic community. Since 2008, a dedicated biennial workshop series on ‘Simultaneous combination of spectroscopies with X-ray absorption, scattering and diffraction’ has been organized.^1^

Several synchrotron crystallography endstations supporting the *in situ* combination of spectroscopy and X-ray diffraction measurements have been constructed so far (Sakai *et al.*, 2002 ▶; Carpentier *et al.*, 2007 ▶; Pearson *et al.*, 2007 ▶; Royant *et al.*, 2007 ▶; Ellis *et al.*, 2008 ▶; Davies *et al.*, 2009 ▶; Owen *et al.*, 2009 ▶; Stoner-Ma *et al.*, 2011 ▶; Allan *et al.*, 2013 ▶; Cohen *et al.*, 2013 ▶). In addition to the instruments constructed by the beamline work groups, user groups have brought micro-spectrophotometers to beamlines and mounted them temporarily for combined spectroscopy–crystallography experiments (Hadfield & Hajdu, 1993 ▶; Beitlich *et al.*, 2007 ▶). The first working prototype of a micro-spectrophotometer at the macromolecular crystallography beamlines of the Swiss Light Source, the SLS-MS (Owen *et al.*, 2009 ▶), featured, as a novelty, a so-called on-axis geometry, with collinear X-ray and optical axes, as well as reflective coupling objectives.

With the SLS-MS, for the first time on-axis UV/Vis absorption and fluorescence spectroscopy at an MX beamline became possible. Further, in proof-of-principle experiments, the potential for on-axis Raman spectroscopy was demonstrated on a frozen drop of cyclohexane, *i.e.* a highly concentrated small molecule sample (Owen *et al.*, 2009 ▶). The focus during development of the SLS-micro-spectrophotometer version 2 (in short, MS2) was set toward enabling resonance Raman and non-resonance Raman spectroscopy also on crystalline protein samples, whose scatterer density is several orders of magnitude lower. This major increase in sensitivity compared with the previous instrument now allows Raman spectra acquisition even on difficult-to-measure protein crystals. Also, great care was taken to improve instrument stability and ease of alignment.

Additionally, with the current version of the micro-spectrophotometer at beamline X10SA, the original concept of the spectroscopic layout was kept, but the actual implementation redone from ground up, retaining of all optical components only the reflective Schwarzschild objectives. With these changes both the excitation and the detection efficiency could be significantly increased, thereby now also fully enabling resonance Raman and non-resonance Raman spectroscopy.

Each of the *in crystallo* spectroscopic methods has specific strengths. The newly implemented Raman spectroscopy modes, as vibrational spectroscopy methods, are particularly suited for the identification of ligands and chemical species, for example, by assignment of specific vibrational bands *via* difference Raman spectroscopy. A typical use case example is the determination of ligand active-site interactions in soaking experiments (Katona *et al.*, 2007 ▶; Kovaleva & Lipscomb, 2008 ▶; Carey *et al.*, 2011 ▶). Resonance Raman spectroscopy additionally exploits the enhancement of the Raman transition probability in resonance or near-resonance with an optical transition. The specificity of the enhancement then directly supports the assignment of vibrational bands related to chromophores and their direct environment (Siebert & Hildebrandt, 2008 ▶).

In the context of optical spectroscopies at a synchrotron-based experiment, an interesting aspect is the dual use of spectroscopy in both controlling the intended and also in avoiding the unwanted effects of the X-ray dose received by the sample. All of the spectroscopic modes can probe the effect of X-ray-induced photophysics in intended exposures (Schlichting *et al.*, 2000 ▶; Kühnel *et al.*, 2007 ▶; Adam *et al.*, 2009 ▶; Hersleth & Andersson, 2011 ▶; He *et al.*, 2012 ▶) and also detect the unwanted effects of the dose accumulated during MX data collection (radiation damage) (Meents *et al.*, 2007 ▶; Carpentier *et al.*, 2010 ▶; Hersleth & Andersson, 2011 ▶; Rajendran *et al.*, 2011 ▶). While the study of the photophysical effects lends itself to specialized studies, the monitoring of radiation damage is indispensable for a wide range of MX experiments. Provided the spectroscopic experiment can be sufficiently facilitated to be conducted by inexperienced users, in particular the radiation damage monitoring can be not only attractive to users but rather become a quasi-requirement for the validation of X-ray diffraction results (Hersleth & Andersson, 2011 ▶; He *et al.*, 2012 ▶; Merlino *et al.*, 2013 ▶). In that regard, the deposition of spectroscopic results alongside the diffraction data in the Protein Data Bank (PDB) has been proposed (Garman & Weik, 2011 ▶; Orville *et al.*, 2011 ▶).

The specific strengths of the different spectroscopic modes have been well reviewed for the fields of structural enzymology (Pearson *et al.*, 2004 ▶; De la Mora-Rey & Wilmot, 2007 ▶; Pearson & Owen, 2009 ▶), temperature-dependent macromolecular crystallography (Weik & Colletier, 2010 ▶), and the mapping of radiation damage and X-ray-induced photophysics (Garman & Weik, 2011 ▶).

The general applicability of the different spectroscopic modes differs strongly. Both UV/Vis absorption and fluorescence spectroscopy require the presence of one or more chromophores in the sample under investigation. Analysis of the presence of colored chromophores in structures deposited in the PDB until June 2010 yields approximately 11000 structures containing at least one colored co-factor (Stoner-Ma *et al.*, 2011 ▶), showing the broad applicability for the described methods. The same criterion also applies to resonance Raman spectroscopy, relying on the strong enhancement of the Raman transition moment upon excitation close to an optical dipole transition.

Non-resonance Raman spectroscopy, in contrast, does not require the presence of a chromophore and therefore in principle opens all non-chromophore-containing samples to spectroscopic investigation. Owing to the absence of the chromophore-specific selectivity, in non-resonance Raman spectroscopy the complete molecule contributes to the spectroscopic signal. To selectively detect changes due to, for example, a chemical reaction therefore imposes a requirement of high signal-to-noise data in the non-resonance Raman mode (Carey *et al.*, 2011 ▶). Strong reduction of sample-intrinsic fluorescent background signals is a central motivation for non-resonance Raman excitation in the near-infrared (NIR) regime and the Raman capabilities of the MS2 were thus extended to the NIR.

Several instrumentation upgrades to the infrastructure of SLS beamline X10SA further increase the instrument’s versatility. Since January 2010, a Pilatus 6M area detector (Dectris Ltd, Baden, Switzerland) has been installed at the beamline, allowing the acquisition of complete diffraction data sets within sub-minute timescales, *i.e.* fast enough to obtain structures of intermediates with lifetimes in the minute range (Regis Faro *et al.*, 2011 ▶). Upon request, a He cryogenic system can be installed instead of the standard N_2_ flow system to reach temperatures down to 10 K.

Last but not least, the online spectroscopy facility is being complemented by an offline SLSpectroLab facility with a separate full goniometer and micro-spectrophotometer set-up for preparatory measurements and measurements not requiring X-rays.

All developments leading to the MS2 instrument have been included in the design of a final version, the MS3, which is currently being commissioned. This new instrument is integrated into the newly designed endstation of beamline X10SA and remains always online. The main difference between the MS2 and the MS3 is a greatly improved performance of the imaging branch owing to custom-designed objectives. A greater working distance renders the MS3 compatible with the sample-mounting robot. Lastly, the design was modularized to support rapid changes between the spectroscopic modes without requiring unmounting of the sample. The MS2 is mounted at the offline facility for continuous off-line experiments accessibility.

## 2. Instrumentation at SLS beamline X10SA

Several key improvements to the design of the first version of the SLS micro-spectrophotometer, SLS-MS (Owen *et al.*, 2009 ▶), were implemented to achieve the coupling efficiency required for Raman spectroscopy experiments and thereby enabled full multi-mode capability. While the basic concept of the layout has remained the same, all optical components of the instrument were optimized, with the sole exception of the high-magnification Schwarzschild objectives (Newport, 15× magnification, *f* = 13 mm, back focal length = infinite, NA = 0.4) and the drilled mirror used to deflect the visible light away from the X-ray axis. The changes led to improvements of the coupling efficiency of the excitation and detection branches, to an increase of the spectral bandwidth and to better overall stability and repeatability of the set-up.

For some of the upgrade iterations of the MS2, relative efficiency improvements can readily be determined. For example, by choosing a wavelength-specific dichroic edge filter instead of a broadband beamsplitter to couple the excitation and collection branches, an improvement of coupling efficiency by approximately a factor of four was achieved (§2.2.3). Further improvements were realised by the exchange of crucial spectrometer components, with similar efficiency gains for each of the design changes such as the implementation of reflective coupling optics (§2.2.5), optimized light guides and filter positioning, or the NIR optimized CCD detector (§2.2.7).

As an alternative to direct instrument figures of merit, achievable signal-to-noise figures for standard samples under defined experimental conditions can be determined. With their commercial availability and published crystallization protocols, the lysozyme and myoglobin examples in the experimental section of this article can serve this purpose. To avoid uncertainties due to the crystal morphology, measurements of frozen solutions in crystallographic loops, *i.e.* thin films, are a viable alternative.

In an effort to extend the bandwidth of the instrument into the UV domain down to below 250 nm, all coupling in the illumination and detection branches of the MS2 is now performed with reflective optical elements instead of glass lenses. As a direct benefit thereof, chromatic aberrations are avoided without resorting to compound lenses. A remaining but unavoidable limitation is the protein crystals’ sample morphology (*i.e.* surface properties, scatterer density and shape), causing extensive Rayleigh scattering in the UV domain, and artifacts (*e.g.* stray light, back reflections or distortion by the nitrogen gas stream) introduced by the suboptimal sample environment (Kessler, 2006 ▶). A known approach to overcome the limited UV performance is the utilization of backscattering measurement geometry rather than a transmissive one. Since such a set-up requires an integrating sphere around the sample, however, it is not compatible with the crowded beamline environment. For Raman and fluorescence spectroscopy, the collection efficiency is limited by the numerical aperture of the objectives, owing to the space limitations of crystallographic endstations. An approach to significantly increase the collection efficiency is the inclusion of a spherical mirror opposite the collection objective (Kamagata *et al.*, 2012 ▶). In the presented set-up, such a mirror could be installed alternative to or alongside the UV/Vis illumination.

While the SLS-MS required a full 8 h shift for the set-up and alignment of the instrument at the beamline, the full procedure, including alignment of the Raman mode, can now be completed in less than 3 h with a build-back time of down to 1 h.

### 2.1. On-axis geometry

The chosen on-axis configuration provides extensive control over the relative arrangement of the irradiated and probed sample volumes, both their relative size and position, through the coaxial arrangement of X-ray and optical beams. As laid out in more detail in §4.1 below, there are clear application scenarios both for an X-ray beam diameter larger than the spectroscopic beams and *vice versa*.

With an off-axis geometry, in contrast to on-axis configurations, overlapping the paths of the X-ray beam and the spectroscopic probes can be difficult or even impossible, depending on the axes’ relative angles. Optimized off-axis sampling geometries with both X-ray and spectroscopic axes perpendicular to the goniometer axis can in principle provide perfect overlap of the sampling volumes, however, still under the limiting conditions of serial data collection and intermittent reorientations (Orville *et al.*, 2011 ▶; Cohen *et al.*, 2013 ▶). An off-axis set-up including a multi-axis goniometer would also fulfill this criterion. Any other off-axis geometries require a careful matching of crystal and beam sizes to achieve a full overlap, thereby introducing strict requirements on samples and reducing experimental options (Carpentier *et al.*, 2007 ▶).

In general, for quantitative dose estimates, crystals larger than the X-ray beam pose a problem for all experimental geometries. During a diffraction data collection on a sample larger than the X-ray beam, with un-irradiated portions of the crystal successively brought into the beam by the ω rotation, different parts of the crystal receive large differences in dose. In the estimation of the maximal dose for radiation-induced effects, this is obviously a problem also for on-axis set-ups and a measurement of a still crystal can then be considered only a conservative estimate. The RADDOSE team has quantitatively modeled the spatial distribution of dose in a crystal to treat this effect for some typical use cases in macromolecular crystallography (Zeldin *et al.*, 2013 ▶).

Apart from the assured overlap of optical probe and X-ray axes, on-axis spectroscopy brings with it further advantages. In resonance Raman spectroscopy, where sampling depth is often very restricted by the limited penetration depth of the excitation laser due to a high absorption coefficient of the chromophore, by choosing a backscattering geometry it can be assured that vibrational information is definitely sampled from an X-ray-irradiated volume.

On-axis geometry also facilitates the kinetic study of X-ray-induced reactions with high time resolution. For example, kinetic UV/Vis absorption spectroscopy in a sacrificial measurement is the perfect tool for precise determination of the total acceptable dose for obtaining unreduced structures of metal-center-containing proteins. If some boundary conditions such as a flat-top X-ray illumination and a sufficiently low absorption are met, the dose becomes deposited evenly in the sample along the optical path. Kinetic measurements allow then to measure precisely the dose required for an X-ray-induced process to occur and to design a diffraction experiment accordingly.

Last but not least, the co-axial imaging provides a direct and unambiguous observation of the probed crystal morphology. Taken together, these features demonstrate how on-axis geometry avoids many systematic errors encountered in geometries with perpendicular axes arrangements. In an on-axis set-up, the illumination lamp downstream of the sample prevents the acquisition of absorption spectra during diffraction data acquisition. Owing to its strong orientation dependence, the acquisition of a spectrum requires a return to the reference orientation, however, so this is not a limitation for practical applications. Other instrumentation difficulties to be resolved with on-axis set-ups, such as a slightly lower numerical aperture due to the drilled optics to pass the X-ray beam, are more than compensated for by these principal advantages.

### 2.2. Micro-spectrophotometer layout

**Figure 1:**
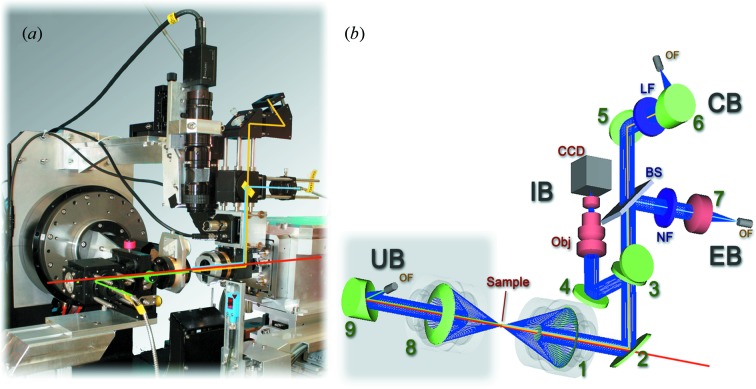
(*a*) The MS2 mounted on beamline X10SA, shown in UV/Vis absorption mode with the illumination Schwarzschild objective moved in for measurement. Red: X-ray beam path; green: illumination light path; blue: laser excitation path; yellow: signal detection light path. (*b*) Schematic representation of the branches, light path and optical components in the MS2. Naming as discussed in the text: IB, imaging branch; CB, collection branch; EB, excitation branch; UB, UV/Vis illumination branch; 1–9, optical elements; OF, light guide fiber coupling; BS, beamsplitter; LF, laser line filter; NF, notch filter.

The layout of the MS2, shown in Fig. 1 ▶, consists of four main components: the imaging (IB), collection (CB) and excitation (EB) branches upstream, and the motorized UV/Vis illumination (UB) branch downstream of the sample. On the upstream side, following the drilled collection objective (1), the light is diverted away from the X-ray axis by a drilled 45° mirror (2) and then distributed between the three branches.

#### 2.2.1. Imaging branch

To be able to monitor the sample and align the probing volume on the sample, an on-axis microscope is integrated into the MS2. The sample is imaged through the same Schwarzschild objective used for focusing the spectroscopic branches (1 in Fig. 1*b* ▶). The light is projected into the imaging branch by use of either a pellicle beam­splitter, which allows for continuous sample observation, or alternatively by a flippable mirror at the same position (3). The latter is less convenient for the user since the mirror has to be flipped in and out of the beam path every time the sample is imaged. For crystalline samples, however, it is not possible to implement a proper spectroscopic reference arm, since the reference spectra are typically measured either in air or in the buffer solution adjacent to the crystal. Absorption spectroscopy on small crystals using a fixed pellicle beamsplitter is therefore sensitive to artifacts due to thin-film interference. Transmission and reflection factors of pellicle beamsplitters are wavelength dependent and highly sensitive to the incident angle of the incoming light. Therefore, with crystals acting as lenses or prisms, the slight difference in the incidence angles between the case when the sample is out of the light path (reference) and in the light path (absorbance) can lead to artifacts in the resulting absorption spectrum. This is mostly observed for small crystals, where the incidence angles probed by the illumination beam are typically steeper than for large crystals. To both avoid these artifacts and ease the alignment of the instrument, a custom-made flippable beamsplitter instead of a mirror will be implemented in the next version of the MS.

For the alignment of the imaging branch, an adjustable mirror parallel to the flippable mirror is used (4). By thus decoupling the components, the alignment of the imaging branch with respect to the X-ray beam is greatly facilitated.

The sample camera optics itself consists of a video objective (Obj; 1A1HB, 1:3.9–32, *f* = 75 mm, TAMRON, Japan) and a color CCD-camera (CCD; GRAS-14S5C-C, Point Grey, USA). Overall, the imaging system has a magnification of 5.8, and a field of view of 1.4 mm × 1.1 mm. For coarse sample alignment and as an additional aid for the alignment of the optical beams, a top-down camera with a larger field of view and a variable magnification is installed.

#### 2.2.2. Excitation and collection branches

For all spectroscopic modes the signal is collected through the Schwarzschild objective also used for imaging (1). The collected light is then transmitted past the diverting elements (3; BS) onto an adjustable 45° mirror (5). This mirror is used primarily for alignment of the collection branch relative to the other branches. Optionally, for Raman spectroscopy, the residual excitation laser line is suppressed here by an edge filter (EF). In contrast to notch filters, edge filters are available with steeper edges and higher absorption and are therefore preferred for Stokes–Raman spectroscopy. For focusing into the optical fiber to the spectrograph (OF) an off-axis parabolic mirror is employed (6).

For spectroscopic modes requiring light excitation of the sample with a laser or a lamp, the light is coupled into the excitation branch (EB) of the spectrophotometer *via* a light guide (OF) using a collimating lens (7). For Raman spectroscopy, sidebands to the main laser line are filtered out using a wavelength-dependent interference laser line filter (LF).

#### 2.2.3. Balance between excitation and collection

The light is deflected towards the sample by a beamsplitter (BS). By varying this element it is possible to influence the balance between the excitation intensity and the transmitted signal in the Raman and fluorescence modes. If a choice for a single beamsplitter for all applications and excitation wavelengths has to be made, in the absence of photo-induced damage to the sample, the optimal ratio between reflection and transmission (*R*/*T*) for the beam-splitter is 1.0, since both the Raman and the fluorescence signals are proportional to the excitation intensity. A choice lower than 1.0, favoring transmission of collected light over excitation, could be used to reduce the light-induced effects on the sample. The overall coupling from excitation to sample and back to the collection branch can be greatly increased by the choice of wavelength-specific dichroic beamsplitters (*T* > 0.9 for the Raman signal) instead of a broadband beamsplitter (*T* ≃ 0.5), to obtain both high reflection at the excitation wavelength and high transmission closely above the excitation wavelength. In the ideal case this increases the signal by a factor of four.

#### 2.2.4. UV/Vis illumination branch

Since UV/Vis absorption measurements are performed in transmissive geometry, an illumination lamp has to be positioned 180° from the collection objective. In an X-ray crystallographic beamline environment, the illumination branch (UB) therefore has to be moved in and out of the X-ray path to avoid interference with the diffraction experiment. This is achieved by utilization of three motorized linear stages which are also used for alignment of the second objective. The branch itself consists of a second, identical, Schwarzschild objective (8) coupled *via* another off-axis parabolic mirror (9) to the incoming light guide (OF) from the remote light source.

#### 2.2.5. Reflective optics

**Figure 2:**
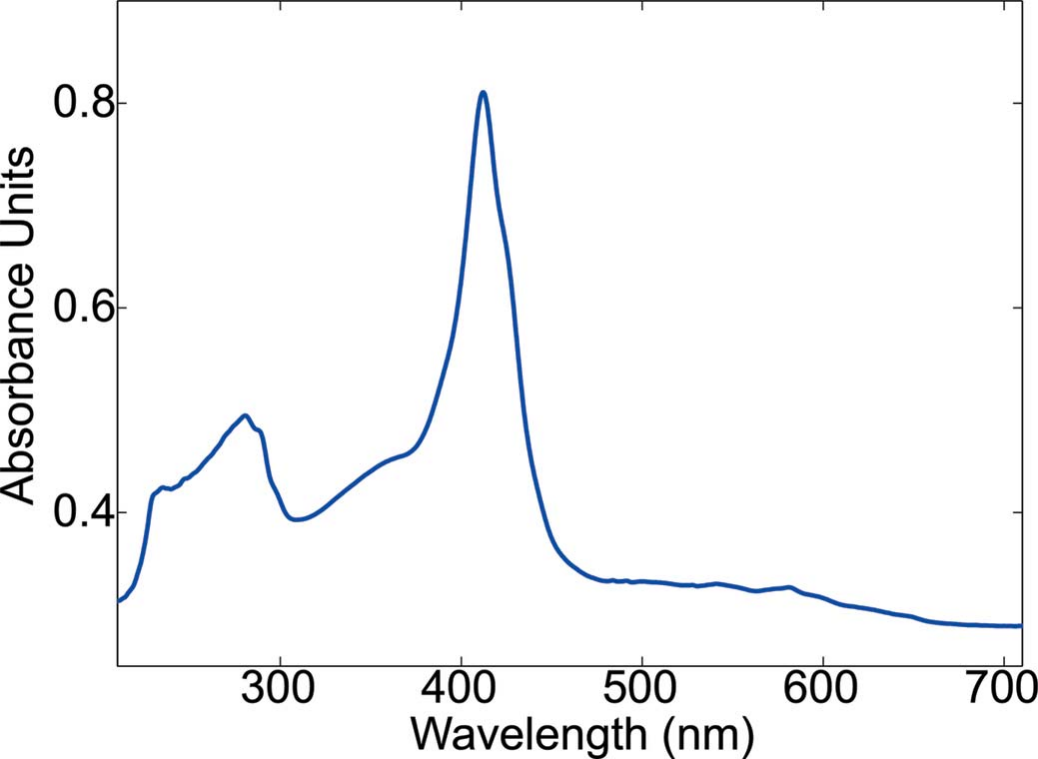
UV/Vis absorption spectrum of a plate-like crystal (50 µm × 20 µm × 10 µm) of partially reduced horse heart myoglobin. To minimize morphological artifacts, the crystal was rotated along ω to yield best absorption spectra. Acquisition time was 100 × 2.6 ms with an entrance slit width of 100 µm, using a 300 lines mm^−1^ grating and a UV-optimized EMCCD detector. The wavelength range in the UV is accessible down to at least 250 nm.

All glass lenses of the SLS-MS with the exception of the excitation branch were replaced by reflective optical elements. The main advantage of reflective optics compared with transmissive lenses lies in its transmission efficiency over the whole spectral domain used in spectroscopy on biological samples, from UV to NIR. A second advantage is the absence of chromatic aberrations which occur in glass or quartz lenses. UV/Vis absorption spectra can now be collected down to wavelengths of 250 nm (Fig. 2 ▶). The collimating optics used to couple the light guides to the illumination and the collection branch have been implemented with off-axis parabolic mirrors with a focal length of 34 mm. Optimal coupling to the optical fibers was achieved by matching the numerical aperture of the focusing optics to that of the fibers.

An example demonstrating the full bandwidth of the current instrument, a typical absorption spectrum of a horse heart myoglobin crystal, is shown in Fig. 2 ▶.

#### 2.2.6. Light sources

The SLSpectroLab facility at the SLS offers a broad range of illumination sources to cover the requirements of the different modes and of the different biologically relevant samples. Two different light sources are available for UV/Vis absorption experiments: a high-power Xenon arc lamp (CLX500, Zeiss, Germany) and a combined deuterium/tungsten lamp (BDS130, BW*Tek, USA) to extend the usable spectral range in the UV domain from 350 nm to well below 250 nm.

To verify that the UV performance is not limited by insufficient illumination, a test measurement with a high UV flux laser-driven light source (LDLS; EQ-99FC, Energetiq, USA) was performed (data not shown). This only marginally improved the UV performance but instead introduced photochemical artifacts in the sample. Among other effects, heavy ozone generation was observed, which leads to a high absorbance peak below 280 nm (Hearn, 1961 ▶).

The lasers available for Raman and fluorescence spectroscopy are listed in Table 1 ▶. The lasers at 405 nm, 473 nm and 785 nm are fiber-coupled and tunable in power. The 532 nm laser is coupled by a lens into an optical fiber mounted on a kinematic holder. The coupling loss mostly depends on the fiber coupling lens and the lens–fiber alignment, with the loss in the fiber itself smaller than 5% as stated by the manufacturer and confirmed by open-beam coupling tests (data not shown). During set-up of an experiment, the effective laser power reaching the sample is measured with a power meter and typically lies between 1/5 and 1/10 of the output power.

#### 2.2.7. Spectrograph

With the addition of a second spectrograph, the MS2 set-up now opens the possibility to switch between two different spectroscopic modes without time-consuming recalibration of the spectrograph and therefore perform complementary investigations at the same position on the same crystal sample. This makes the MS2 a true multi-mode instrument as systematic errors from comparing results from different crystals or from sequential measurements can be completely avoided.

Both spectrographs are Czerny–Turner instruments (Shamrock SR-303i-A, Andor Technologies, Ireland) and are equipped with different grating selections and CCD detectors. Entrance slit widths of the spectrographs can be tuned from 10 µm to 1.2 mm.

One spectrograph is optimized for data acquisition in the visible domain – UV/Vis absorption and resonance Raman spectroscopy with excitation at wavelengths shorter than 600 nm. It is equipped with an electron-multiplying CCD camera (Newton EM-CCD DU-970N, 1600 × 200 array of 16 µm × 16 µm pixels, Andor Technologies, Ireland). Its three gratings (150 lines mm^−1^, 600 lines mm^−1^ and 2400 lines mm^−1^) are blazed at 300 nm and mounted on a motorized turret for on-the-fly changing.

The second spectrograph is optimized for non-resonance Raman spectroscopy at longer wavelengths, in particular excitation at 785 nm in the NIR domain. This spectrograph is equipped with a NIR-optimized deep-depleted CCD camera (Newton DU-920-N-BR-DD, 1024 × 255 array of 26 µm × 26 µm pixels, Andor Technologies, Ireland) and its motorized turret contains three gratings optimized for long wavelengths (600 lines mm^−1^ blazed at 500 nm, 600 lines mm^−1^ blazed at 1000 nm, and 1200 lines mm^−1^ blazed at 1000 nm). The deep-depleted CCD chip technology avoids etaloning effects at NIR frequencies at the expense of a somewhat higher dark current and providing lower spectral resolution due to the larger pixel size.

The detectors utilize air-cooled Peltier elements for cooling the CCD arrays to 193 K, to significantly reduce dark currents for low-photon-count measurements like Raman spectroscopy. At this temperature the readout noise of the CCD electronics becomes the dominant noise source.

### 2.3. Off-line SLSpectroLab

An off-line laboratory, the SLSpectroLab, directly adjacent to the beamline X10SA, has been set up and is being opened for user operation. The laboratory is a replication of the online set-up with a full crystallographic goniometer and a cryogenic cooling system (CryojetXL by Oxford Instruments, Agilent Technologies). This laboratory offers the opportunity for users to either prepare an upcoming online experiment by evaluating the quality of their crystals especially for Raman spectroscopy, or carry out any experiments for which the X-ray beam is not required. The off-line laboratory also contains the UV/Vis light sources, the excitation lasers and the spectrographs to which the MS2 is coupled *via* optical fibers of 20 m length running from the laboratory to the experimental hutch of the beamline.

### 2.4. Operation

#### 2.4.1. Alignment

**Figure 3:**
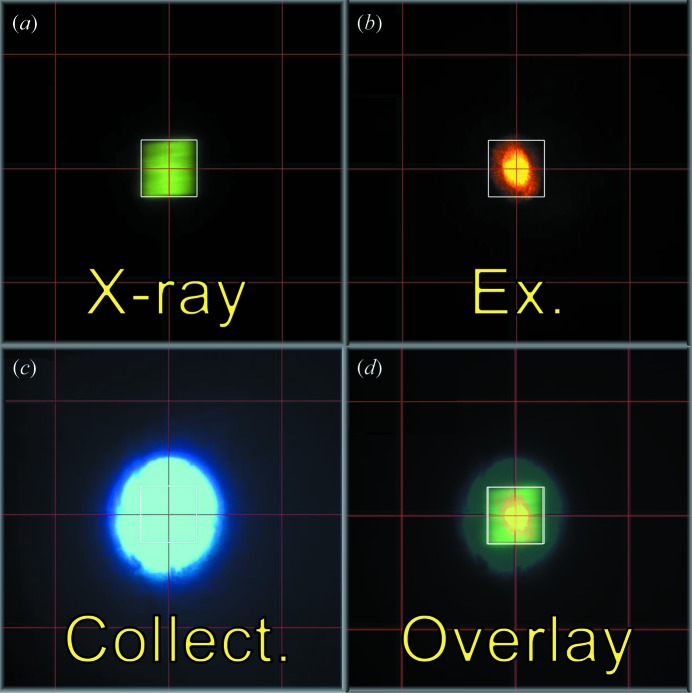
Typical beam sizes for an experiment with the X-ray beam larger than optical beams, imaged on a Ce:YAG scintillator screen mounted at the sample position. (*a*) X-ray beam (100 µm × 100 µm). (*b*) 785 nm laser excitation spot (50 µm). (*c*) Spectroscopic collection area in the focal plane, imaged by reverse illumination through the collection branch (200 µm). (*d*) Computer-generated overlay of all spots.

The on-axis geometry considerably facilitates the alignment of the spectroscopic axes with the X-ray beam, since the beam overlap can be monitored both by the on-axis and the top-down camera. The motorized scintillator screen at the sample position, which is part of the standard diffractometer set-up, serves to both image the X-ray beam and the spectroscopic illumination beams. The use of indirect aids such as pinholes and extra intensity monitors can thereby be completely avoided and instead one can directly rely on the coaxiality of all involved axes and the imaged beam waists (see Fig. 3 ▶).

Before mounting the MS2 at the diffractometer, the collimation of the imaging branch is ensured. After mounting, it is aligned with respect to the stationary X-ray axis. The imaging branch is then used as a reference to which all other axes are aligned. The goniometer axis is imaged in the sample camera *via* a centered tungsten needle. The foci of the excitation and collection branches are adjusted with respect to that of the imaging branch by translating their respective fiber holders. The beam waists can be monitored by fogging the cryostream and observing it *via* the top-down camera. To ensure lateral beam overlap, the flippable mirror of the imaging branch is temporarily replaced by a pellicle beam splitter, which is thin enough not to displace the beam paths. This enables the observation of the excitation and reverse-illuminated collection branch beams and their overlap with the X-ray beam imaged on a Ce:YAG scintillator screen.

Depending on the type of experiment, the X-ray beam can be adjusted to be smaller, of the same size or larger than the spectroscopic probe (see §4.1) below. In the same vein, the size of the laser excitation spot can be tuned from 25 µm to 100 µm by using optical fibers of different core diameter (from 50 µm to 200 µm) and the appropriate collimating lenses.

#### 2.4.2. Calibration of the spectrographs

The wavelength axes of the spectrographs are calibrated using the peaks of an Hg lamp spectrum. For Raman spectroscopy, a second cross calibration is performed by collecting the Raman spectrum of a standard sample like a Si wafer, frozen cyclohexane or 4-acetamidophenol. The reference values for the line assignments were obtained from McCreery (2000 ▶, 2011 ▶).

## 3. Materials and methods

### 3.1. Crystallization

Horse heart myoglobin and hen egg-white lysozyme (HEWL) were purchased from Sigma-Aldrich. They were used without further purification and crystallized by the vapor diffusion technique using the hanging drop method.

Horse heart myoglobin was crystallized by equilibrating 3 µl of protein at 10 mg ml^−1^ with the same volume of the reservoir solution, consisting of 3.4 *M* ammonium sulfate and 0.1 *M* tris buffer at pH 8.0. Cryoprotection was performed by soaking the crystals for 10 s in a solution of 2.6 *M* ammonium sulfate, 0.1 *M* tris buffer at pH 8.0 and 25% glycerol.

HEWL was crystallized by mixing 2 µl of protein at 30 mg ml^−1^ with the same volume of reservoir solution consisting of 0.8 *M* NaCl and 100 m*M* acetate buffer at pH 4.5. Crystals were cryoprotected by soaking them for 10 s in the mother liquor solution supplemented by 25% glycerol.

Derivative crystals of HEWL were prepared using the protocol described by Pompidor *et al.* (2010 ▶). The crystals were back-soaked and cryoprotected with two consecutive transfers of 15 s in the cryoprotectant solution identical to the reservoir solution supplemented by 25% glycerol and then flash-cooled directly in the N_2_ cold stream.

### 3.2. Diffraction data collection

The reflective Schwarzschild objective used for both sample visualization and focusing of spectroscopic beams has a working distance of 24 mm, compared with the 35 mm of the normal on-axis microscope of beamline X10SA. Therefore, the beam-shaping devices, *i.e.* a variable aperture and Mo tube scatter guard, are replaced by a shorter Mo tube collimator (0.5 mm outer diameter, 0.35 mm inner diameter) between the Schwarzschild objective and the sample to absorb background X-ray radiation due to air scattering. Air scatter from the open X-ray beam path inside the reflective objective is suppressed by inserting a drilled aluminium plate in front of the objective during X-ray exposure. The next version of the instrument, the MS3, will use a custom-designed objective featuring an internal collimator tube and a working distance of 35 mm, obviating the use of an attenuator cover and enabling the use of the standard beam-shaping devices.

### 3.3. Spectroscopic data acquisition and control software

#### 3.3.1. Data collection

The spectrographs and the spectroscopic CCD cameras are controlled by the SOLIS control suite (Andor Technologies, Ireland). Control of the optical peripherals, for example lamps and laser shutters, is provided through dedicated Experimental Physics and Industry Control System MEDM control panels (http://www.aps.anl.gov/epics/).

To ensure user and equipment safety, all motor movements at the beamline environment are controlled *via* a dedicated Experiment State Controller (ESCAPE) and workflow engine (Ebner, 2012 ▶).

#### 3.3.2. Data post-processing

Post-processing of Raman spectroscopic data is usually unavoidable due to the strong X-ray-induced fluorescence background masking the typically one to two orders of magnitude weaker Raman signal. To eliminate bias as much as possible, we employ automated baseline reduction routines based on a modified asymmetric least-squares algorithm (Peng *et al.*, 2010 ▶; Liland *et al.*, 2010 ▶).

Data post-processing beyond the capabilities of the *SOLIS* software is handled by dedicated MATLAB (Mathworks, USA) scripts and GUIs, comprised in the APE-toolbox (Advanced file Processing Environment; in-house development, to be published).

## 4. Results and discussion

The measurements in the following section were selected with a focus on demonstrating the new and upgraded features of the SLS micro-spectrophotometer. Beginning with an experimental quantification and validation of the optimized on-axis sampling geometry, the fluorescence mode is demonstrated, followed by the Raman modes, both under resonant and non-resonant conditions.

### 4.1. Beam profile mapping: X-ray *versus* spectroscopic beams

**Figure 4:**
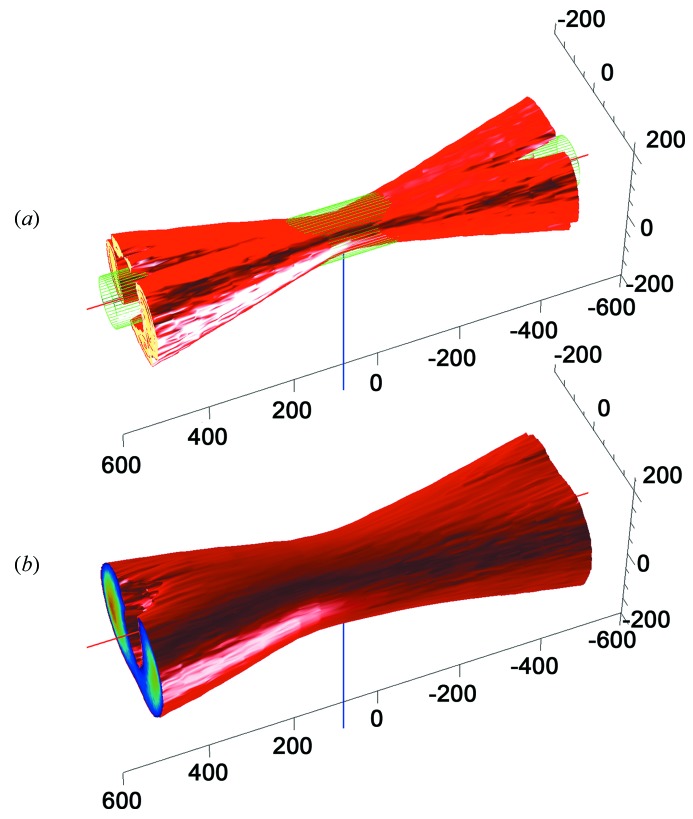
Three-dimensional reconstruction of beam profiles of (*a*) the excitation branch of the MS2 (coupling *via* 100 µm fiber) and (*b*) the collection branch (coupling *via* 400 µm fiber). Illumination intensities were measured on a CCD detector array mounted perpendicular to the X-ray axis. Intensity images were recorded every 50 µm in the range from 0.5 mm upstream to 0.5 mm downstream of the sample position, and the illumination volume was reconstructed using MATLAB. The red line shows the X-ray axis with the green mesh depicting the approximate size of the X-ray beam (100 µm); the blue line marks the sample position. Left is downstream and right upstream. Beam sizes depicted here are identical to the configuration shown in Fig. 3 ▶. From these mappings the sample volumes with full overlap of X-ray and spectroscopic beams can be determined.

To characterize the overlap between the X-ray beam and the spectroscopic probe area provided by the MS2’s on-axis configuration, the optical beam profiles were determined along the X-ray axis at the sample position. A CCD camera (EO-1312C, 4.65 µm × 4.65 µm pixel size, 1280 × 1024 pixels, 8-bit resolution, Edmund Optics) mounted on a three-axis stage was aligned at the sample position perpendicular to the X-ray beam and then stepped through the collection volume. Illumination intensity on the individual images was determined and used to reconstruct the spectroscopic volumes using the MATLAB software (The Mathworks, USA). Fig. 4 ▶ shows the beam profiles of the collection branch and excitation with optical fibers of 100 µm and 400 µm, respectively.

The size of the X-ray beam itself was measured by stepping a scintillation screen through the beam and measuring the spot size with the normal on-axis microscope (data not shown). Consistent with the beamline’s horizontal beam divergence of 0.6 mrad, the size of the X-ray spot over the whole range was measured to be almost constant for both the focused (50 µm × 15 µm) and a defocused beam (100 µm × 100 µm). Taken together, these results allow a precise characterization of the sampling volumes encountered in different modes of the on-axis configuration. On the one hand, the superimposition of the waist of the excitation laser with a defocused X-ray beam of 100 µm × 100 µm (Fig. 4 ▶, top) demonstrates that spectroscopy strictly probes an irradiated part of the sample over a distance of 300 µm along the X-ray beam axis. Most of the crystals match the maximum thickness of 300 µm. In the case of larger crystals, to achieve full overlap, compared with the set-up in Fig. 4 ▶ one has to increase the X-ray beam size relative to the laser. Therefore one can choose either a smaller core diameter for coupling the laser source in the excitation branch (50 µm) or a wider defocused X-ray beam than 100 µm × 100 µm. On the other hand, by focusing the X-ray beam (50 µm × 15 µm) and selecting a coupling fiber with a large core diameter (200–400 µm), X-ray diffraction can probe strictly an illuminated volume of the crystal.

For example, monitoring X-ray-induced photoreduction would require an X-ray beam diameter larger than that of the spectroscopic probe. Spectroscopic information thus obtained from the core of the X-ray-irradiated area provides information only about molecules that contributed to the diffraction signal. In contrast, the structural characterization of a laser-excited state would require the opposite beam diameter ratio, such that the X-ray diffraction probes the illuminated volume.

In general, the beam spot sizes have direct implications for the accessible sample sizes. The different modes of the micro-spectrophotometer have different restrictions on sample sizes. For absorption spectroscopy, minimal crystal sizes of approximately half the illumination spot size can still be measured, *i.e.* 20 µm. The maximal crystal size is given by the thickness corresponding to an optical density (OD) of around 2, and therefore strongly sample-dependent. For resonance Raman spectroscopy, the minimal sample size strongly depends on the resonance enhancement and co-factor arrangement; in general, in an on-axis configuration larger crystals give better signals. Crystals around 50 µm in size have successfully been measured with the MS2, with an excitation spot size of 50 µm. Under non-resonance conditions, the sample thickness is crucial; samples smaller than 100 µm in all dimensions become impractical for reasonable sampling times under 10 min.

### 4.2. Luminescence spectroscopy on HEWL derivative crystals prepared with a europium complex

Luminescence spectroscopy, comprising the detection of either fluorescence or phosphorescence photons, is a technique requiring the presence of a fluorophore or a phosphorescent center in the protein crystals. Compared with Raman spectroscopy the method is less challenging in terms of detection efficiency. The luminescence quantum yields (from 1 down to lower than 10^−6^) are commonly higher than even resonance Raman quantum yields [below 10^−5^ (Siebert & Hildebrandt, 2008 ▶)]. Therefore the main task for a given luminescence experiment is to provide the proper dedicated light source and filters.

As an example we show the luminescence of a HEWL derivative crystal prepared with a luminescent europium complex, Na_3_[Eu(DPA)_3_].6H_2_O, where DPA stands for pyridine-2,6-dicarboxylate (dipicolinate). The complex was demonstrated to be valuable in the preparation of high-phasing-power derivative crystals. For some derivative crystals prepared by co-crystallization, the complex, binding at the interface between protein molecules in the crystal, can act as a crosslinking agent and improve the diffraction quality compared with native crystals (Pompidor *et al.*, 2010 ▶). Derivative crystals were soaked twice in a cryoprotectant solution identical to the mother liquor supplemented by 25% glycerol but without the lanthanide complex.

Owing to their electronic configuration, where 4*f* electrons are shielded by 5*s* ones, lanthanide ions exhibit interesting luminescence properties with sharp emission bands and large Stokes shifts.

**Figure 5:**
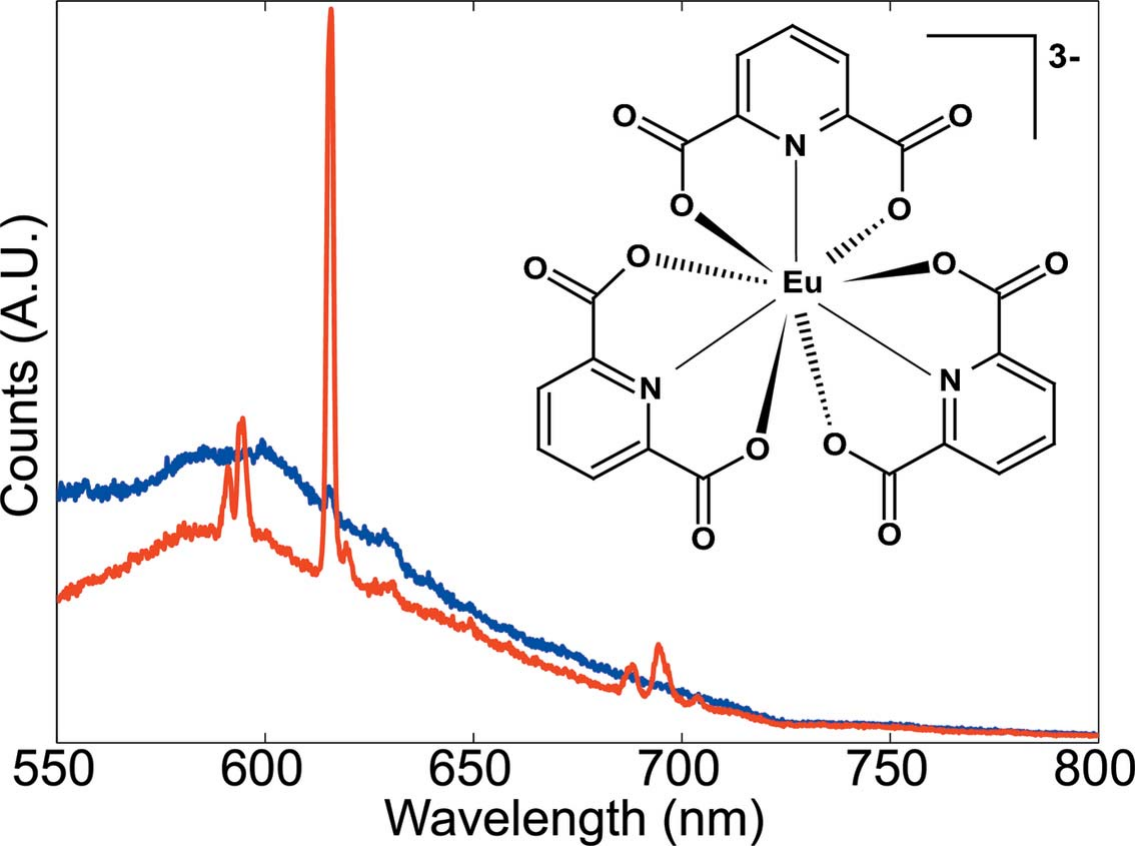
Luminescence spectra of a HEWL derivative crystal prepared with Na_3_[Eu(DPA)_3_] complex (red) and of the mother liquor from the surrounding crystal (blue) upon illumination with a 532 nm laser with a power density of 26 mW mm^−2^ and acquisition times of 100 × 0.27 s and 20 s, respectively.

Luminescence of the europium ion was excited at 532 nm in the parity-forbidden ^7^ *F*_1_ → ^5^ *D*_4_ transition with an extinction coefficient estimated to be 0.015 l mol^−1^ cm^−1^. The luminescence spectrum (Fig. 5 ▶) exhibits all the emission lines of the Eu(III) (Binnemans *et al.*, 1997 ▶). In the derivative crystals, the europium complex is bound at the interface between molecules. Owing to the back-soaking, the luminescence signal stems only from the complexes trapped between lysozyme molecules in the crystal. The control spectrum was collected in the drop surrounding the crystal, to ensure that the luminescence originated only from the europium complexes bound in the crystal, corresponding to a concentration of 150 m*M*. With two back-soakings, the liquor surrounding the crystal is almost completely freed from the europium complex which is then only present in the crystal, as assessed by the luminescence spectrum of the solution which exhibits only a small peak corresponding to the most intense emission line of Eu(III) at 615 nm. The luminescence provides an easy and fast method for detecting the complex binding in the derivative crystals prepared by co-crystallization. Successful derivatization can then be quickly checked prior to the more time-consuming anomalous diffraction data collection and processing.

This experiment demonstrates the capabilities of the MS2 to perform luminescence spectroscopy on solution samples, with a typical chromophore concentration found in protein crystals. With an excitation in a forbidden transition, *i.e.* with a low extinction coefficient, the luminescence signal was collected within less than 30 s, highlighting the high detection sensitivity of the instrument.

### 4.3. Resonance Raman spectroscopy on horse heart myoglobin crystals

Horse heart myoglobin (Mb), like most heme proteins, is particularly sensitive to photoreduction by photoelectrons (Beitlich *et al.*, 2007 ▶; Meents *et al.*, 2007 ▶; Hersleth & Andersson, 2011 ▶). With the strong Soret band absorption in the blue, it is a prime target for the resonance Raman technique, to follow its oxidation state marker bands and thereby quantify the reduction process. As a typical case we show a kinetic study of the photoreduction of ferric myoglobin Mb(III) into ferrous Mb(II) by resonance Raman spectroscopy.

**Figure 6:**
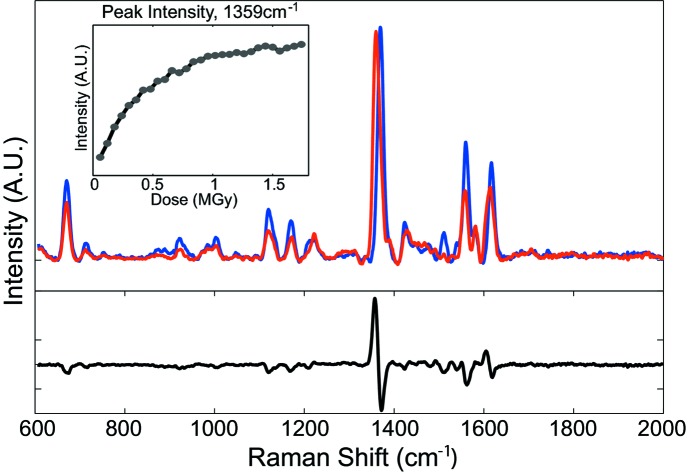
Resonance Raman kinetic measurement of X-ray-induced photoreduction in a myoglobin crystal. The difference spectrum between the unexposed spectrum (blue) and a spectrum after exposure to 1.5 MGy (red) shows reduction of the heme in the X-ray beam as a peak shift from 1371 cm^−1^ to 1359 cm^−1^. Owing to the high sensitivity of the instrument, the reaction could also be followed in real time, showing the dose-dependence of the reduction (inset). Power at the sample was 15 mW and acquisition time for each spectrum was 10 s.

A rosette-shaped cluster of Mb(III) crystals (50 µm × 50 µm × 50 µm) was cryoprotected and frozen directly in the N_2_ gas stream. The crystals were exposed to X-rays at low flux [80 × 10^9^ photons s^−1^ at 12.4 keV, corresponding to a dose rate of 3 kGy s^−1^ calculated for a 100 µm × 100 µm beam with RADDOSE (Paithankar *et al.*, 2009 ▶)] while resonance Raman spectra at 405 nm excitation wavelength were collected every 10 s for 10 min (Fig. 6 ▶). For the success of the measurement, it is essential to ensure that the sample is not influenced by the laser exposure. Therefore a first kinetic experiment was performed without any X-ray irradiation, to ascertain that the laser power did not induce any photoreduction of Mb(III) into Mb(II), as would be indicated by the shoulder appearing around 1360 cm^−1^ [ν_4_ shifts upon reduction (Spiro, 1985 ▶)]. No laser-induced reduction could be observed. Even with the short acquisition time of 10 s, the quality of the spectra allows the identification of all the vibrational bands reported in the literature. The 2400 lines mm^−1^ gratings blazed at 300 nm provide an excellent spectral resolution (3 cm^−1^) and allow the whole region of interest to be collected in a single spectrum (from 600 cm^−1^ to 2000 cm^−1^).

After baseline subtraction, the difference Raman spectrum between the first spectrum (zero dose) and the last one shows the peak shifts occurring upon photoreduction from a ferric to ferrous state. The ferric state (zero dose) spectrum exhibits all the vibrational modes typical of an aquo six-coordinated high-spin heme (6cHS) [1480 cm^−1^ (ν_3_), 1513 cm^−1^ (ν_38_), 1543 cm^−1^ (ν_11_), 1562 cm^−1^ (ν_2_) and 1582 cm^−1^ (ν_37_)] reported in earlier studies [Table 2 ▶ and Liu *et al.* (1990 ▶), Takahashi *et al.* (1994 ▶), Smulevich *et al.* (1995 ▶), Hu *et al.* (1996 ▶), Lu *et al.* (2005 ▶), De Sanctis *et al.* (2007 ▶)]. The presence of the intense band at 1618 cm^−1^ corresponding to the ν(C=C) vinyl stretching modes is in agreement with a 6cHS form.

The differences found in Raman peak wavenumbers are all in the range of our instrument resolution (3 cm^−1^ for the chosen slit and grating combination) as well as the 5 cm^−1^ cited by Takahashi *et al.* (1994 ▶) and De Sanctis *et al.* (2007 ▶). The deviations from the literature peak positions could be attributed to the difference between the samples: physical and chemical parameters as well as the sample state (crystal and solution). Takahashi and co-authors noticed peak shifts in the high-frequency domain between Mb(III) at pH 6.5 and at pH 10.5 and attributed these shifts to a partial conversion from high spin to low spin occurring at high pH. Therefore, with our initial spectrum being unambiguously the 6cHS form, pH can be ruled out as the reason for the observed differences.

The main effect of X-ray-induced photoreduction of Mb(III) into Mb(II) on the Raman spectra is the shift of the ν_4_ and the ν(C=C) bands from 1371 cm^−1^ to 1359 cm^−1^ and from 1618 cm^−1^ to 1615 cm^−1^, respectively. These results are in agreement with the study of Takahashi and co-workers who reduced the Mb from the ferric to the ferrous form using sodium dithionite. The time courses of the two positions of the ν_4_ band at 1371 cm^−1^ and 1359 cm^−1^ indicate that the cryo­radiolytic reduction is complete within a dose of 1.5 MGy. Beitlich and co-workers reported a dose of 2.0 MGy by studying the same process by UV/Vis absorption (Beitlich *et al.*, 2007 ▶). This difference can be explained by the uncertainties in the determination of the X-ray flux density distributions.

The demonstrated high sensitivity of the resonance Raman mode of the MS2 makes this mode a powerful alternative to UV/Vis absorption spectroscopy in detecting X-ray-induced photoreduction, particularly in cases where the crystal thickness makes absorption spectra acquisition impossible.

### 4.4. Non-resonance Raman spectroscopy

In contrast to resonance Raman spectroscopy, non-resonance Raman spectroscopy is potentially applicable to any crystal of biological macromolecules as it does not require the presence of a chromophore. However, owing to the lack of any resonance enhancement, all Raman active vibrational modes of the macromolecule are observed simultaneously, causing overlap of the bands and making assignment more difficult. High spectroscopic detection sensitivity is therefore required to overcome the lower Raman transition moment and to apply more advanced methods such as difference spectroscopy. To minimize the background signal from protein intrinsic fluorescence, excitation wavelengths are preferably shifted to the NIR domain. To demonstrate the performance of the MS2 in non-resonance Raman spectroscopy, Raman spectra have been collected with excitation at 785 nm on HEWL crystals.

**Figure 7:**
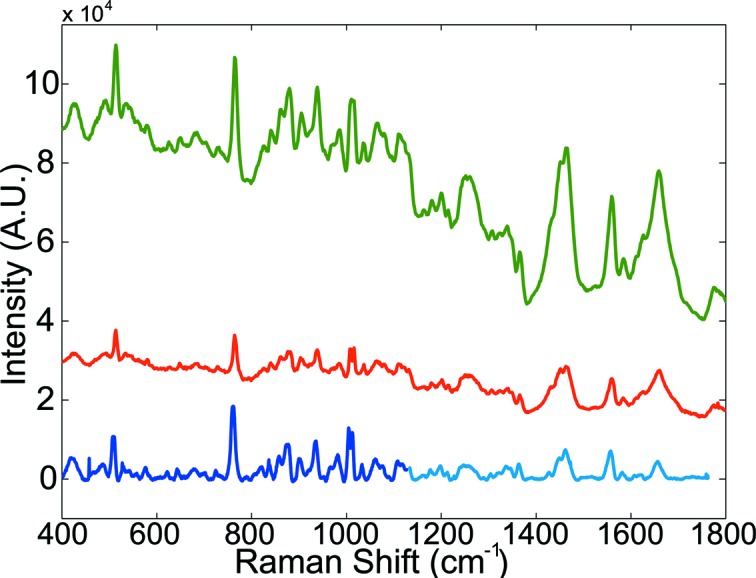
Non-resonance Raman spectra of a HEWL crystal using different grating and slit settings. Green: 600 lines mm^−1^ grating, 100 µm slit size with 100 s acquisition time; red: 600 lines mm^−1^ grating, 10 µm slit size and 240 s acquisition time; blue: 1200 lines mm^−1^ grating, 100 µm slit size with 180 s acquisition time. Spectra were acquired with excitation at 785 nm. The last spectrum was manually stitched together from two separate acquisitions (400–1120 cm^−1^ and 1040–1760 cm^−1^) to extend to a comparable spectral range. For this spectrum a basic baseline correction was applied. All other spectra show raw data. Power at the sample was 160 mW.

Fig. 7 ▶ shows a non-resonance Raman spectrum of a HEWL crystal with an acquisition time of 180 s. The vibrational modes reported in the literature (Kudryavtsev *et al.*, 1998 ▶) can be clearly assigned: disulfide bond (510 cm^−1^ and 525 cm^−1^), methionine (725 cm^−1^), the Trp side-chains (543 cm^−1^, 759 cm^−1^, 875 cm^−1^, 1012 cm^−1^ and 1361 cm^−1^), the Tyr side-chains (834 cm^−1^, 855 cm^−1^ and 1208 cm^−1^), the Phe side-chain residues (1004 cm^−1^ and 1196 cm^−1^) and acidic residues (940–980 cm^−1^).

Owing to the high quantum efficiency of the deep depleted CCD camera in the NIR domain compared with the EMCCD model, non-resonance Raman spectra of high quality can be collected with very short acquisition times, typically from 1 to 5 min for a complete spectrum, and a few seconds to detect the largest peaks to optimize crystal orientation. With gratings optimized for NIR spectroscopy (1200 lines mm^−1^, blazed at 1000 nm), Raman signals can be collected from 400 cm^−1^ to 1100 cm^−1^ with a spectral resolution of 4.65 cm^−1^ (100 µm slit size). With these settings, the whole spectral domain of interest for biological macromolecules, from ∼400 cm^−1^ to 2000 cm^−1^, can be covered in only two acquisitions. Using the 600 lines mm^−1^ grating blazed at 1000 nm, the Raman spectrum even includes the whole domain of interest with a spectral resolution of 5.2 cm^−1^ or 11.2 cm^−1^, using 10 µm or 100 µm slit sizes, respectively. The larger slit width results in an approximate tenfold increase in photon detection, while only reducing the spectral resolution by a factor of two, so that this is the preferred setting unless peaks very close together have to be resolved (*e.g.* peaks around 1010 cm^−1^ in Fig. 7 ▶). The selection of the gratings and the size of the entrance slits of the spectrograph as well as the tunable intensity of the 785 nm laser make the non-resonance Raman set-up in the NIR domain highly versatile. Depending on the experiment, a focus can be put either on fast data acquisition and signal-to-noise ratio or on spectral resolution.

**Figure 8:**
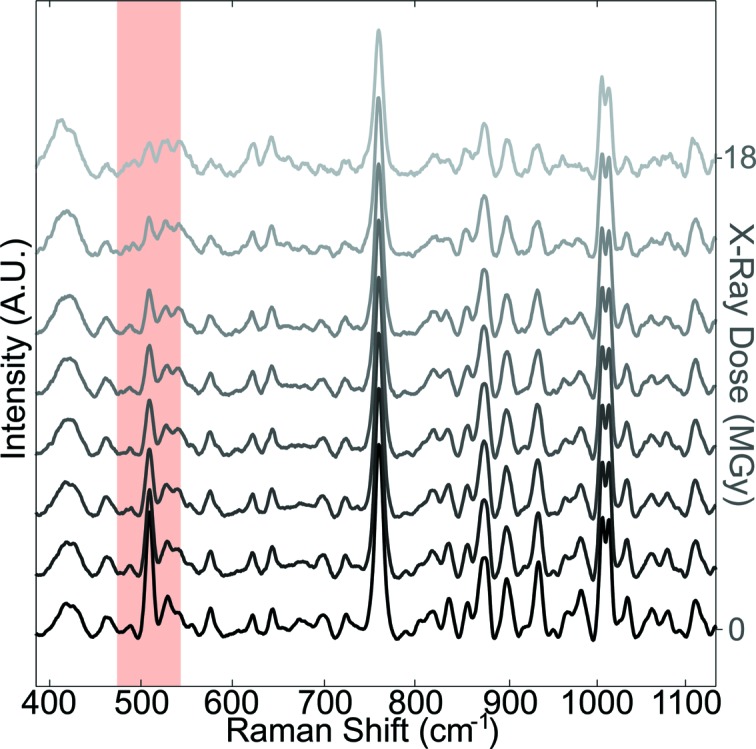
Non-resonance Raman time-series showing the X-ray-induced breakage of disulfide bonds (510 cm^−1^, red line) in HEWL in quasi real-time (λ_exc_ = 785 nm). Spectra are ordered with lowest X-ray dose at the bottom, final dose (18 MGy) at the top. Power at the sample was 160 mW and acquisition time for each spectrum was 180 s.

Both the on-axis geometry and the high efficiency of the MS2 in the NIR domain are of great interest for the study of radiation damage processes. In Fig. 8 ▶, non-resonance Raman spectra collected on the same HEWL crystal with an increasing X-ray dose are shown. The spectra were baseline subtracted, but not additionally scaled, since the peak at 759 cm^−1^ of the Trp did not show significant variation. This was also suggested in a work on the dose-dependent decay of the S—S bond stretching Raman band at 510 cm^−1^ combined with crystallography (Carpentier *et al.*, 2010 ▶). The comparison of the successive Raman spectra shows the decay of the peaks at 830–860 cm^−1^ and 940–980 cm^−1^ which could be assigned to further specific radiation damage effects such as the de­hydroxylation of tyrosine residues and the decarboxylation of acidic residues, respectively.

This observation of specific radiation damage effects demonstrates that the performance of the non-resonance Raman mode of the MS2 in the NIR opens up the possibility for further investigations such as, for example, reaction kinetics and ligand binding studies (Carey *et al.*, 2011 ▶).

## 5. Conclusion

With the upgrade to full multi-mode operation, the SLS micro-spectrophotometer MS2 now also supports Raman and resonance Raman operation. With its increased bandwidth down to wavelengths of 250 nm, the option to rapidly switch experimental modes and a set-up time of less than 3 h, the scope of supported experiments has been considerably expanded. Its unique on-axis configuration avoids systematic errors from incomplete overlap of spectroscopic sampling volume and X-ray-irradiated area and generally facilitates spectrophotometer alignment. The new micro-spectrophotometer makes beamline X10SA attractive both for broader uses like dose determination to avoid specific radiation damage effects and also, in combination with the off-line SLSpectroLab and the fast Pilatus 6M hybrid pixel detector, for a specialized field like, for example, kinetic enzymology. The facility has been open for user operation since 2009, with successful collaborations with over 15 external user groups (Antonyuk & Hough, 2011 ▶; Hersleth & Andersson, 2011 ▶; Regis Faro *et al.*, 2011 ▶; He *et al.*, 2012 ▶; Owen *et al.*, 2012 ▶; Merlino *et al.*, 2013 ▶). The final upgrade, MS3, which is currently being commissioned, includes and improves on the advances of the MS2 instrument presented here and, most importantly, can remain always online at the beamline

## Figures and Tables

**Table 1:** Available laser light sources and specifications

Model	Wavelength (nm)	Linewidth (nm)	Power before fiber (mW)	Typical power at sample (mW)
Omicron Laserage LDM405.400.CWA	405	<0.02	500	50
CrystaLaser CL473-150-0	473	0.1	150	30
Cobolt Samba 500	532	<0.00001	500	55
Coherent Innova 302-C	647	<0.07	>400	40
Bayspec MNLS MiniLite	785	0.08	700	160

**Table 2:** Raman bands observed for a myoglobin crystal, with assignments from Hu *et al.* (1996 ▶)

Band assignment	Mb(III) (cm^−1^)	Mb(II) (cm^−1^)
ν_7_	673	673
γ_11_ γ_5_	717	715
ν_15_	755	755
γ(=C_*b*_H_2_)*s* and ν_46_	925	927
γ(C_a_H=)	987	987
ν_45_	1003	1006
γ(=C_*b*_H_2_)*as*	1050	1050
γ(=C_*b*_H_2_)*as*	1093	1093
ν_5_	1123	1122
ν_14_	1134	1133
ν_30_	1171	1174
ν_13_	1215	
CH_2_ twist	1223	1224
γ(C_*a*_H=)	1316	1316
ν_4_	1371	1359
ν_28_	1427	1430
δ(=C_*b*_H_2_)	1448	
ν_3_	1480	1471
ν_38_	1513	1513
ν_11_	1543	
ν_2_	1562	1561
ν_37_	1582	1584
ν(C_*a*_=C_*b*_)	1618	1615

## Footnotes

1 The most recent workshop, CSX2012, took place in 2012 at the ETH in Zurich, Switzerland, and the next instance is organized by Brookhaven National Laboratory, USA, in 2014.
